# Guidelines for genetic counselling in ATTR amyloidosis

**DOI:** 10.1186/1750-1172-10-S1-I20

**Published:** 2015-11-02

**Authors:** Jorge Sequeiros

**Affiliations:** 1 IBMC – i3S and ICBAS, Univ. Porto, Porto, Portugal

## Definition

Genetic counselling is “a process of communication that deals with the occurrence, or risk of occurrence, of a (possibly) genetic disorder in the family” (ASHG, 1975; EuroGentest guidelines). It involves an attempt, by appropriately trained persons, to help patients and their families to (1) understand the medical facts of the disease; (2) appreciate the contribution of heredity and risks of recurrence in relatives; (3) understand the consultands’ options to deal with those risks, including all their reproductive options; (4) use this genetic information in a personally meaningful way that promotes health, minimizes psychological distress and increases personal control; (5) choose the course of action that seems appropriate to them and act in accordance with that decision; and (6) make the best possible adjustment to the disease or the genetic risks (modified by EuroGentest Unit 3 from Frazer, AJHG 1974; Biesecker & Peters, AJMG 2001; Harper, 2012).

## Guidelines

Genetic counselling must always begin with the establishment or documentation of a clinical and genetic diagnosis in a proband, followed by counselling of relatives at risk. In the context of presymptomatic testing (PST), i.e., in healthy relatives at high-risk for a late-onset monogenic disease with high penetrance, as ATTR amyloidosis, a protocol of pre- and post-test genetic counselling needs to be offered; and be accompanied by psychosocial evaluation and support, their purpose being to assess motivations for testing, explore decision-making processes, test coping mechanisms, predict risk of adverse emotional reactions, prepare psychosocial support, identify values and family dynamics and reinforce social support networks. Family’s experience (late vs. early-onset) is critical. PST should begin with relatives at higher risk (cascade testing). Minors should not be tested, as they might lose their autonomy and suffer discrimination. In the context of diagnostic testing, neurological symptoms must be evident, so that the genetic laboratory does not perform an inadvertent PST. In the context of prenatal diagnosis (PND) or of preimplantation genetic diagnosis (PGD), both parents must be involved in the counselling protocol, one of them must be affected or a presymptomatic carrier, with a TTR mutation previously identified in the family; in addition, for PND, there must be a clear motivation for termination of pregnancy if the foetus is a mutation carrier.

## Conclusions

Availability of therapies and ongoing clinical trials may be a (false) motivation for increased adherence to PST; however, this is not relevant for treatment (will change when preventive measures or presymptomatic treatment become possible). ATTR amyloidosis, though potentially treatable, is still currently incurable (mutation is always present and heritable). Genetic counselling is always mandatory.

